# *PyQCstrc.ico*: a computing package for structural modelling of icosahedral quasicrystals

**DOI:** 10.1107/S1600576721005951

**Published:** 2021-07-16

**Authors:** Tsunetomo Yamada

**Affiliations:** a Faculty of Science, Department of Applied Physics, Tokyo University of Science, 6-3-1 Niijuku, Katsushika-ku, Tokyo 125-8585, Japan

**Keywords:** quasicrystals, higher-dimensional crystallography

## Abstract

The atomic structure of quasicrystals (QCs) is described as a section of a higher-dimensional structure that consists of a periodic arrangement of occupation domains (ODs). Determination of the shape of ODs and their partitioning is crucial in the structural analysis of QCs. However, owing to the complicated shape of ODs, building the initial structure model requires a great deal of time and effort. Thus, a computer program for building structure models of QCs is needed. Presented here is a Python3 package for structure modelling of icosahedral QCs.

## Abstract 2

*PyQCstrc.ico* is a computer package for building a 6D structure model of icosahedral quasicrystals with the Python3 programming language.

## 1. Introduction   

Quasicrystals (QCs) are long-range-ordered materials that generate self-similar diffraction patterns related to their scale incompatible with translational symmetry (Shechtman *et al.*, 1984 ▸; Levine & Steinhardt, 1984 ▸). The atomic structure of QCs can be described as a 3D section of *n*-dimensional (*n*D) periodic structures (*n* > 3). The *n*D structure consists of ‘occupation domains’ (ODs) or atomic surfaces whose geometrical shapes are defined in the (*n* − 3)D complementary space called perpendicular space (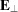), perpendicular to the 3D real space, called parallel space (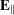) (see *e.g.* Yamamoto, 1996 ▸; Janssen *et al.*, 2007 ▸). The ODs form 2D surfaces or 3D solids for dihedral (*n* = 5) or icosahedral (*n* = 6) QCs, respectively.

Structural analysis of QCs can be executed by utilizing the software package *QUASI* developed by Yamamoto (2008 ▸). This contains several programs which can be utilized for the direct method (*lodemac*) (Takakura *et al.*, 2001 ▸), structure refinement (*qcdiff*), generation of atomic positions in 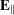 (*qcstrc*), and generation of Fourier and maximum entropy method (MEM) maps (*qcmem*) *etc*. However, a tool for building the initial structure model in the *n*D space has been lacking, which means scientists have to put a great deal of work into building the initial models. To overcome this difficulty, the *PyQCstrc* library for the Python3 programming language was developed, with the aim of providing a tool for building *n*D models. In this article, I present the *PyQCstrc.ico* package in the library, which is dedicated to QCs with an icosahedral symmetry.

This article is organized as follows. In Section 2 I briefly describe the ODs in the 6D models for two 3D quasiperiodic tilings. In Section 3 I describe the Python package *PyQCstrc.ico*, which can be used to build and arrange the ODs in a 6D icosahedral lattice. In Section 4 an example is given, and a summary is provided in Section 5.

## 2. Occupation domains   

In the higher-dimensional description of icosahedral quasicrystals (iQCs), the 6D model is described by a 6D icosahedral lattice which is decorated by 3D ODs embedded in 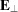. The 6D positional vector 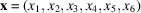 can be represented by using the unit vectors of the 6D icosahedral lattice, 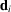 (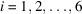) (see Appendix *A* [App appa]). Note that the choice of the unit vectors is arbitrary, and there are several conventions. The most frequently used are the conventions proposed by Cahn *et al.* (1986 ▸), Elser (1985 ▸) and Yamamoto (1996 ▸), and Yamamoto’s is used in the package. The atomic positions are obtained from periodically arranged ODs by taking the intersections with 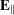. Thus, the shape of ODs and their positions in the 6D space must be derived to build the initial model of the iQCs.

**Figure 1:**
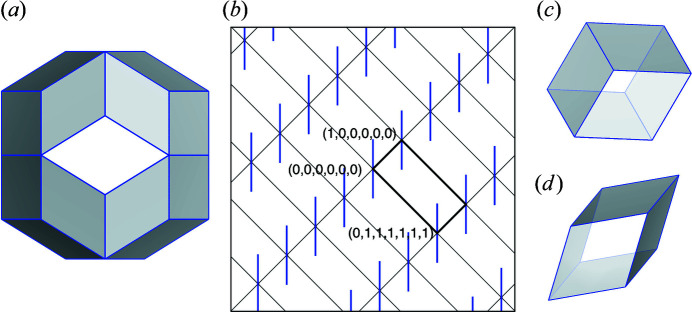
6D structure model of the AKN tiling. (*a*) The rhombic triacontahedron (RT) OD that generates the vertices of the tiling. (*b*) A fivefold section of the 6D structure. Blue bars and black thick lines indicate projection of the RT ODs and 6D unit cell onto the section, and the horizontal and the vertical of the figure are parallel to 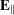 and 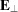, respectively. Two building units of the AKN tiling, (*c*) obtuse and (*d*) acute rhombohedra.

Fig. 1 ▸(*a*) shows the OD that generates the vertices of the 3D Amman–Kramer–Neri (AKN) tiling (Kramer & Neri, 1984 ▸; Duneau & Katz, 1985 ▸; Elser, 1985 ▸, 1986 ▸; Kalugin *et al.*, 1985 ▸). The OD forms a rhombic triacontahedron (RT), which corresponds to the projection of the unit cell of the 6D icosahedral lattice onto 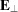, and it is centred at either the origin 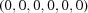 or the body centre 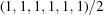, as shown in Fig. 1 ▸(*b*). Since the site symmetry of these positions is 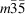, the RT OD can be generated from its asymmetric unit by applying the 120 symmetry elements of 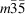. Here, the asymmetric unit forms a tetrahedron, and its Cartesian coordinates in 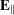 and 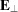 are obtained from 6D vectors 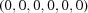, 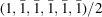, 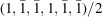 and 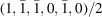 by the projection operator (see Appendix *A* [App appa]). The AKN tiling is composed of two building units, obtuse and acute rhombohedra, shown in Figs. 1 ▸(*c*) and 1 ▸(*d*), respectively.

**Figure 2:**
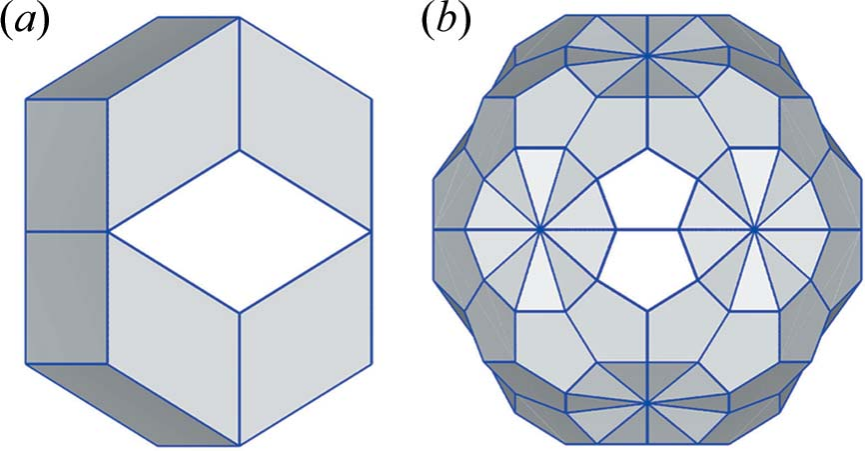
(*a*) Rhombic icosahedron OD that generates the edge centre position of the AKN tiling. (*b*) A truncated RT OD that generates the 12-fold packing sites of the AKN tiling. The truncated RT OD in (*b*) is enlarged by 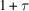, where τ is the golden ratio equal to 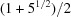.

To decorate the 3D quasiperiodic tiling with atoms, a new OD must be introduced and arranged in the 6D icosahedral lattice. The shape and position of the OD can be derived by utilizing the basic OD for the 3D quasiperiodic tiling. More details about the atomic decorations can be found in the literature (Yamamoto, 1992 ▸, 1996 ▸; Quiquandon *et al.*, 2014 ▸). For example, the edge centre position of the AKN tiling is generated by the rhombic icosahedron (RI) shown in Fig. 2 ▸(*a*), centred at 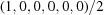. The RI OD is obtained as a common part of two RT ODs at 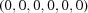 and 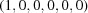, when they are projected onto 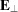 (Yamamoto, 1992 ▸).

The basic OD that is used for a realistic model becomes more complicated. For example, in the cluster-based model of primitive iQCs, the atomic clusters are arranged at the 12-fold packing sites of the AKN tiling (Yamamoto & Hiraga, 1988 ▸; Yamamoto, 1992 ▸; Takakura *et al.*, 2007 ▸; Quiquandon *et al.*, 2014 ▸). The cluster centre positions are generated by the truncated RT OD proposed by Henley (1986 ▸), shown in Fig. 2 ▸(*b*), centred at either 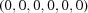 or 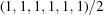, and the relationship between local configurations of the clusters and the partitioning of the OD can be found in the literature (Takakura, 2008 ▸; Takakura & Strzałka, 2017 ▸). The truncated RT can be generated from its asymmetric unit. The atomic decoration of the clusters can be obtained by arranging the basic truncated RT OD in the 6D icosahedral lattice. When the ODs projected onto 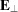 intersect, in many cases the common part forms a concave polyhedron. One of the most difficult parts in building the initial model is related to the calculation of the common part.

## 3. Implementation and availability   

In order to use *QUASI* for structural analysis, each OD must be defined as a set of tetrahedra, and the 6D positional vector **x** whose perpendicular components assign each vertex of the constituent tetrahedra must be known. In the *PyQCstrc.ico* package, all vertices of a tetrahedron forming an OD are defined by 6D coordinates (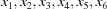), and the value of 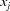 (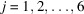) is expressed as 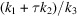, where 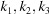 are integers and τ is the golden ratio equal to 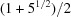.

The Cartesian coordinates in 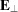 are given by 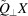, where 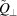 is the lower 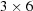 part of the transposed matrix of *Q* [equation (2)] and *X* is a transposed matrix of 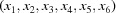. The resulting Cartesian coordinates are represented by a list with integer components when the unit is taken to be 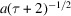. This representation allows one to perform numerical computation free from the influence of rounding error.

The package provides tools commonly used to build the initial structure of iQCs, which include translation and symmetry operations in the 6D space, and intersection operations on ODs in 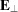. In addition, export and import of the ODs are supported: the ODs can be exported in *VESTA* format (.vesta) and *XYZ* format (.xyz), and these can be visualized by utilizing *VESTA* (Momma & Izumi, 2011 ▸). The latter can be imported so that users can recall their ODs in Python scripts. Moreover, the package supports the *QUASI* formats (.pod and .atm) (Yamamoto, 2008 ▸), as described in the supporting information.

In realistic models of iQCs, the ODs may be concave polyhedra, and their tetrahedralization is not straightforward. In the package, the intersection operation of such ODs is performed by considering every pair of two tetrahedra included in each OD. Because the intersection of two tetrahedra forms a convex polyhedron, its tetrahedralization can be achieved by 3D Delaunay triangulation. Then, the common part is obtained as the union of the resulting tetrahedra. In the case where the common part forms a convex polyhedron, such as the intersection of two convex polyhedra, the above process can be shortened, and the solution is obtained by 3D Delaunay triangulation.

The package is distributed in the Python Package Index (PyPI) software repository (https://pypi.org/project/pyqcstrc/) and is freely available via pip install pyqcstrc. The package requires two libraries, *numpy* (https://numpy.org/) and *scipy* (https://www.scipy.org/), and the Cython programming language (https://cython.org/). Moreover, it is recommended that one uses Python3.7 or greater built with the SSL module, *OpenSSL* (https://www.openssl.org/). The package was developed and tested under macOS (10.14), and it was also tested on other operating systems, including Linux (Ubuntu20.04, Fedora32 and CentOS8) and Windows 10.

## 4. Example   

In this section, the basic usage of the *PyQCstrc.ico* package is described by providing a procedure in a Python script to obtain the RI OD [Fig. 1 ▸(*b*)]. We start with the asymmetric unit of the basic RT OD [Fig. 1 ▸(*a*)]. This forms a tetrahedron whose vertices are assigned 6D coordinates as mentioned before. As seen below, the *j*th component of the 6D coordinates (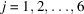), 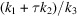, is expressed by utilizing a list [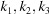]. The RI OD can be calculated in the following way:

(i) Creating the asymmetric unit of the basic RT OD:[Chem scheme1] 
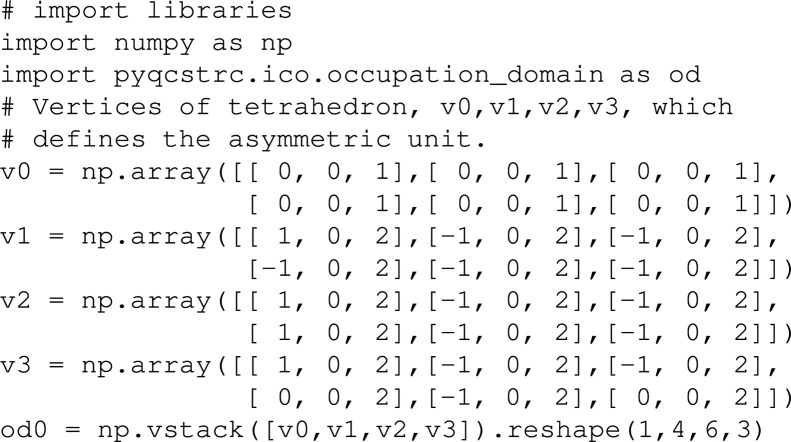


Alternatively, the asymmetric part predefined in a file (rt_asymmetric.xyz) provided in the supporting information can be loaded as od0:[Chem scheme2] 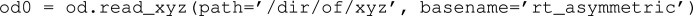


Here, ’/dir/of/xyz’ indicates a directory where the rt_asymmetric.xyz file is located.

(ii) Creating the RT OD from its asymmetric unit by applying the symmetry elements of 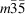 around the origin:[Chem scheme3] 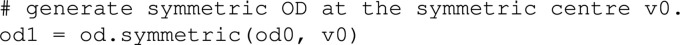


(iii) Creating the RT OD centred at 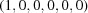:[Chem scheme4] 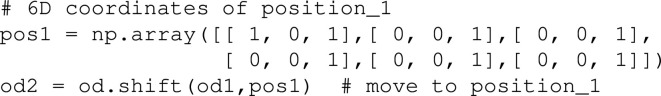


(iv) Intersection operation on od1 and od2:[Chem scheme5] 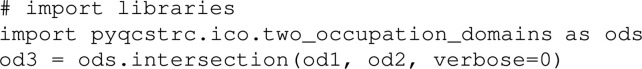


(v) Export the resulting RI OD (od3) in *VESTA* and *XYZ* formats:[Chem scheme6] 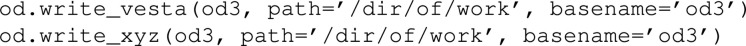


Here, ’/dir/of/work’ indicates a working directory.

(vi) od3 can be imported from the od3.xyz file:[Chem scheme7] 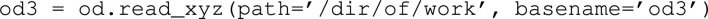


The calculation takes several minutes. To output the progress on the terminal, users can turn on verbose output by specifying an argument ‘verbose’ set to be 1 (verbose = 1) or larger.

Since the RI OD (od3) forms a convex polyhedron, it is tetrahedralizable by 3D Delaunay triangulation. In this case, ods.intersection_convex() can be used, instead of (iv), as follows:[Chem scheme8] 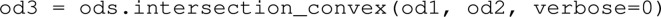


Moreover, the calculation cost can be greatly reduced by considering the asymmetric unit of the position where the solution is located. Here, a portion of od1 inside the asymmetric unit of 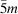 at 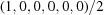 is considered, and obtained as below:[Chem scheme9] 
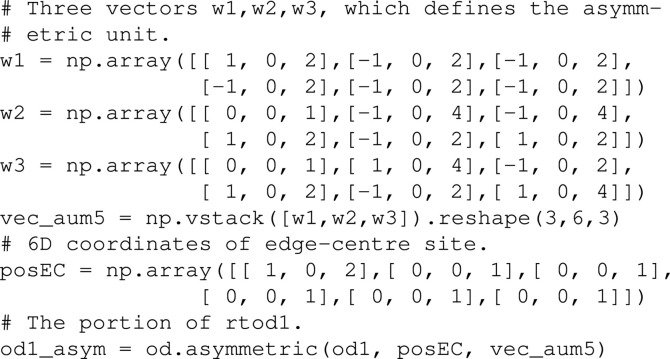


Then, the asymmetric unit of od3 can be obtained by the following:[Chem scheme10] 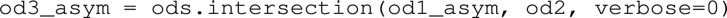


In the above expression, the asymmetric unit of 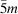 is defined by three vectors 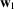, 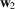 and 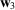. The projection of 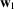 onto 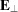 is parallel to a fivefold axis given by the projection of 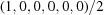, while the projections of 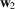 and 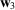 are orthogonal to the fivefold axis. They are on different mirror planes which make an angle of 36°.

## 5. Summary and perspectives   

The *PyQCstrc.ico* package provides tools commonly used to build the initial structure models of iQCs in the Python3 programing language. This is particularly crucial for realistic structure models where the overall shape of ODs is quite complicated. In addition, the package is useful to check the closeness conditions of the ODs and to specify atomic pairs related to phason flips. The latter can be used to compute the short-range-order diffuse scattering, based on the theory proposed by Yamamoto (2010*a* ▸,*b* ▸).

The *PyQCstrc.ico* package is part of the *PyQCstrc* library. Two other packages dedicated to decagonal and dodecagonal QCs are scheduled to be included in the future.

## Supplementary Material

Further details of the software PyQCstrc.ico. DOI: 10.1107/S1600576721005951/tu5010sup1.pdf

Asymmetric unit of occupation domain in Fig.1a (rt_asymmeric.xyz). DOI: 10.1107/S1600576721005951/tu5010sup2.txt

Asymmetric unit of occupation domain in Fig.2b (strt_asymmetric.xyz). DOI: 10.1107/S1600576721005951/tu5010sup3.txt

## Appendix A Coordinate system

The coordinate system used in this study is based on the one described in the literature (Yamamoto, 1996 ▸). The unit vectors of the 6D icosahedral lattice,  (), are written using unit vectors in 3D , , , , and 3D , , , , as

with

where *a* is the lattice parameter of the icosahedral lattice.
