# Supplementary material for: PyQCstrc.ico: a computing package for structural modelling of icosahedral quasicrystals
Source: J Appl Crystallogr. 2021 Jul 16;54(Pt 4):1252–5. doi: 10.1107/S1600576721005951 (PMC8366420; doi:10.1107/S1600576721005951)
Supplement: Supplementary file 1 [file j-54-01252-sup1.pdf]

JOURNAL OF APPLIED CRYSTALLOGRAPHY

Volume 54 (2021)

Supporting information for article:

PyQCstrc.ico: a computing package for structural modelling of icosahedral quasicrystals

Tsunetomo Yamada

1. File format of QUASI (*.pod and *.atm)

In QUASI software, a higher-dimensional structure model is defined in two different files saved in text files with extensions ".pod" and ".atm" [1].

In general, the 6D structure of iQCs consists of the several large ODs centred at high symmetrical positions of the 6D unit cell, and the 6D coordinates are assigned by the 6D positional vector $\mathbf{x}_0$. Each of the ODs can be decomposed into small ODs, and the centres of these are in general apart from the $\mathbf{x}_0$. The shift from the $\mathbf{x}_0$ in the perpendicular space is represented by $\mathbf{x}^i$. In addition, the shape of each small ODs can be defined by specifying 6D coordinates of corner vectors, $\mathbf{e}_j$, of their constituent tetrahedra. Here, the origin of corner vectors $\mathbf{e}_j$ is at $\mathbf{x} = \mathbf{x}_0 + \mathbf{x}^i$.

In QUASI, the shape of each small ODs is defined in the "pod" file, by specifying the corner vectors $\mathbf{e}_j$ of their constituent tetrahedra. In the "atm" file, the users can choose some of the small ODs in the "pod" file and specify their positional vectors, $\mathbf{x}_0$ and $\mathbf{x}^i$. The detailed information of these files is described in the literature [1].

2. How to export .pod and .atm files

By using the *PyQCstruc.ico* package, the users can export vectors, $\mathbf{e}_j$ and $\mathbf{x}^i$, of the ODs to in text files with extensions ".pod" and ".atm", after specifying the vector $\mathbf{x}_0$ and the vertices that define the small ODs. For the shake of simplicity, only the asymmetric unit of the ODs is accepted for this export option. In following, I briefly explain how to export the OD "common_od_asym" which appears in the paper, as an example.

(1) The OD (common_od_asym) is centred at edge-centre position, (1,0,0,0,0,0)/2. For the shake of simplicity, move it to the origin by a shift of (-1,0,0,0,0,0,0)/2 (v4):

```
import numpy as np
import pyqcstrc.ico.occupation_domain as od
v4 = np.array([[-1, 0, 2],[ 0, 0, 1],[ 0, 0, 1],[ 0, 0, 1],[ 0, 0, 1],[ 0, 0, 1]])
common_od_asym = od.shift(common_od_asym,v4)
```

(2) To specify the small ODs, it is convenient to visualize the ODs by using VESTA-3 software [2]. Export the OD in vesta file, and open the generated file (common_od_asym.vesta) in the software:

```
vtx=od.write_vesta(obj = common_od_asym, basename = ' common_od_asym', select = 'podatm')
```

Note that first line returns 6D coordinates (vlst) of the independent vertices of the OD.

(3) Export the 6D coordinates (vlst) in xyz file:

```
od.write_xyz(obj = vtx, basename = ' common_od_asym', select = 'vertex)
```

(4) Once the vesta file generated in (2) is successfully loaded in VESTA-3, the constituent tetrahedra in the OD are visualized. On selection of a ball, "site number" is displayed for the ball.

(5) The users can subdivide the OD in their manner, by specifying "site number" of the vertices of constituent tetrahedra for each small ODs. Each subdivision has to be made so that they have common vertex. In this example, one can subdivide the OD (common_od_asym) into two small ODs. The first and second consist of three and ten tetrahedra, respectively, and these are specified by suing "site number" as following.

Three constituent tetrahedra in the first small OD:

1st tetrahedron, #4, #2, #5, #6,

2nd tetrahedron, #4, #1, #2, #3,

3rd tetrahedron, #4, #1, #2, #5.

Ten constituent tetrahedra in the second small OD:

1st tetrahedron, #9, #1, #2, #5,

2nd tetrahedron, #9, #1, #2, #3,

3rd tetrahedron, #9, #2, #3, #12,

4th tetrahedron, #9, #1, #3, #7,

5th tetrahedron, #9, #3, #7, #11,

6th tetrahedron, #9, #3, #11, #12,

7th tetrahedron, #9, #11, #12, #14,

8th tetrahedron, #9, #8, #11, #14,

9th tetrahedron, #9, #7, #8, #11,

10th tetrahedron, #9, #7, #8, #13.

Note that vertices #4 and #9 are common to all tetrahedra in the first and second ODs, respectively.

(6) Prepare a list (vlst) specifies the above subdivisions. Constituent tetrahedra in each subdivided ODs are represented by a list with integer components beginning with the "site number" of the common vertex, which is followed by a sequence of "site number" for other three vertices, and they are merged into a list (vlst) as below.

```
vlst = [[4,2,5,6,1,2,3,1,2,5],[9,1,2,5,1,2,3,2,3,12,1,3,7,3,7,11,3,11,12,11,12,14,8,11,14,7,8,11,7,8,13]]
```

(7) Export "pod" and "atm" files:

```
EC = np.array([[ 1, 0, 2],[ 0, 0, 1],[ 0, 0, 1],[ 0, 0, 1],[ 0, 0, 1],[ 0, 0, 1]])
vlst=od.read_xyz(basename = ' common_od_asym, select = ' vertex')
od.write_podatm(obj, position = EC, vertices = vtx, vlist = vlst, basename = 'strt_asymmetric')
```

Here, the vector $\mathbf{x}_0$ is specified by 'position' which corresponds to 6D coordinates (EC).

Reference

[1] Yamamoto, A. (2008). *Software package for structure analysis of quasicrystals*. *Science and Technology of Advanced Materials*, **9**(1), 013001.

[2] Momma, K. & Izumi, F. (2011). *Vesta 3 for three-dimensional visualization of crystal, volumetric and morphology data*. *Journal of Applied Crystallography*, **44**(6), 1272–1276.
